# Scanning reflection ion microscopy in a helium ion microscope

**DOI:** 10.3762/bjnano.6.114

**Published:** 2015-05-07

**Authors:** Yuri V Petrov, Oleg F Vyvenko

**Affiliations:** 1 Interdisciplinary Resource Center for Nanotechnology, Saint-Petersburg State University, Ulyanovskaya 1, Saint-Petersburg 198504, Russia,; 2 Faculty of Physics, Saint-Petersburg State University, Ulyanovskaya 1, Saint-Petersburg 198504, Russia

**Keywords:** helium ion microscope, low-angle ion scattering, reflection microscopy, surface imaging, surface morphology

## Abstract

Reflection ion microscopy (RIM) is a technique that uses a low angle of incidence and scattered ions to form an image of the specimen surface. This paper reports on the development of the instrumentation and the analysis of the capabilities and limitations of the scanning RIM in a helium ion microscope (HIM). The reflected ions were detected by their “conversion” to secondary electrons on a platinum surface. An angle of incidence in the range 5–10° was used in the experimental setup. It was shown that the RIM image contrast was determined mostly by surface morphology but not by the atomic composition. A simple geometrical analysis of the reflection process was performed together with a Monte Carlo simulation of the angular dependence of the reflected ion yield. An interpretation of the RIM image formation and a quantification of the height of the surface steps were performed. The minimum detectable step height was found to be approximately 5 nm. RIM imaging of an insulator surface without the need for charge compensation was successfully demonstrated.

## Introduction

Reflection ion microscopy (RIM) is a technique that uses low-angle, scattered ions to form an image of the specimen surface. This technique is similar to reflection electron microscopy (REM) proposed by Ruska in 1933 [1] who realized REM using an angle of 90° between the incident and reflected beams. Later, in the 1950s, a REM technique employing lower incidence angles [2–4] was developed to increase the sensitivity to vertical surface irregularities. A vertical resolution of tens of nanometers was obtained and it was shown that the negative effect of chromatic aberration was also reduced at low incidence angles, yet was still more pronounced in REM as compared to TEM [2,4]. The further development of REM in ultrahigh vacuum conditions allowed imaging of the single atomic steps [5–8] and monitoring of atomic layer-by-layer crystal growth by means of reflection high energy electron diffraction (RHEED) [9]. In the late 1960s, scanning reflection electron microscopy (SREM) was developed [10–11] for the scanning electron microscope (SEM). Chromatic aberration does not appear in SEM because the sample is placed outside of the electron optics. Both REM and SREM require a sufficiently long depth of focus for high quality imaging because of the glancing angle of incidence geometry.

The helium ion microscope is a new scanning microscope with a single atom ion source that was developed in 2006 [12–15]. One of the main features of this device is a large depth of focus provided by a narrow beam divergence angle of about 0.5 mrad [15–16], which is ten times less than the best beam divergence angle possible in SEM. The large depth of focus makes helium ion microscopy (HIM) a very promising tool for scanning reflection microscopy. During the last decade the imaging capabilities of HIM were examined in the field of material science [15,17–23] as well as in biology [15–16,24–26]. Different techniques such as secondary electron energy filtering [27], helium ion channeling contrast [28–30], helium ion transmission microscopy [31], secondary ion mass spectrometry [32–33], and ionoluminescence [34–35] were developed.

In this paper we report on the development of the instrumentation and the analysis of the capabilities and limitations of scanning reflection ion microscopy (RIM) in a helium ion microscope. In the experimental part of our work we describe a configuration of a sample holder developed for RIM and demonstrate first results obtained for standard test samples. In the discussion part a simple theoretical treatment of the image contrast formation in RIM is provided, which is necessary to obtain quantitative information from RIM images.

## Experimental

**Figure 1:**
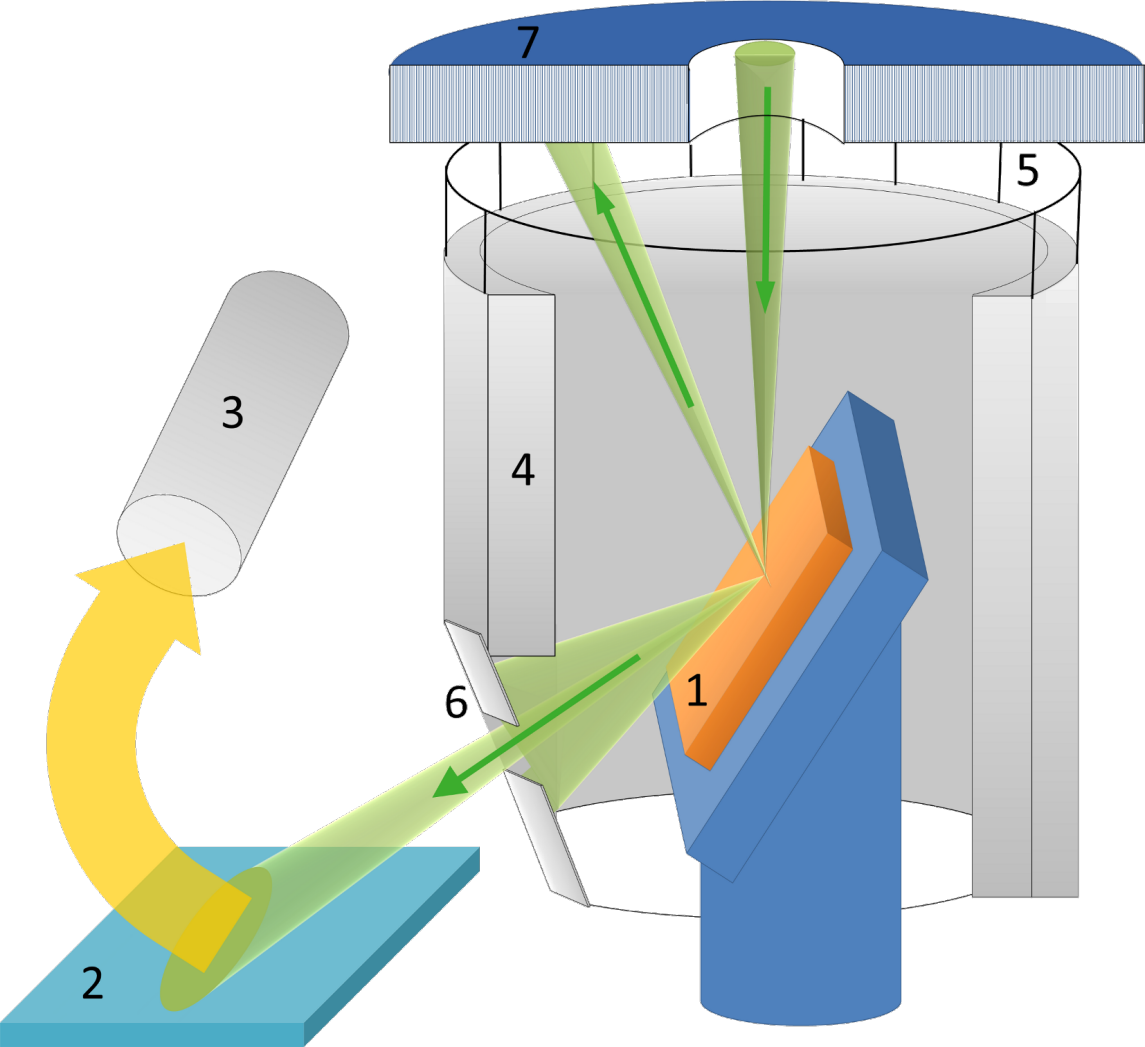
Schematic of the detection of reflected ions in the helium ion microscope. 1 – sample, 2 – Pt-coated surface, 3 – secondary electron detector, 4 – SE grounded shield, 5 – additional SE shield (SE3), 6 – slit diaphragm, 7 – backscattered ion detector.

All investigations were performed in a Zeiss Orion scanning helium ion microscope. The detection of reflected ions (RIs) requires an additional detector installed below the specimen. One of the easiest ways to detect the reflected ions is by their “conversion” to secondary electrons (SEs). Previously, such a RI-to-SE conversion was used for the detection of backscattered electrons in SEM [36] and for scanning transmission mode in HIM [37]. A schematic of the originally developed RI detection system is shown in Figure 1.

The sample (1) was mounted on a stage at a grazing angle relative to the focused helium ion beam. The reflected helium ions were directed to the Pt-coated plate (2) which served as the main RI-to-SE converter. Platinum was chosen because of its high SE yield under He-ion excitation [12]. The SE signal was measured by a conventional Everhart–Thornley (ET) detector (3). The sample was surrounded by grounded metal shields (4 and 5) and was kept under a positive bias of 100 V to exclude the detection of SEs coming from the sample and SEs excited by backscattered ions from the chamber walls and objective lens (by the so-called SE3). The shield (5) is shown as transparent in Figure 1 in order to make the ion beam paths visible. The detection of SEs excited from the conductive sample occurred when the bias was switched off, as the SEs excited from the conductive sample exceeded the signal of the RI-to-SE converter. For insulating samples, the SE signal from the sample could not be detected because of positive charging as will be described further in detail below.

The angular aperture of detected reflected ions was limited by the slit (6) installed under specular reflection conditions. The slit, with the width of about 1 mm, was mounted parallel to the tilt axis of the sample stage at a distance of about 14 mm, which corresponds to an RI detection angular aperture of 4°. Backscattered ions (BSI) were detected by the microchannel plate detector (7). The width of the scanned area was less than tens of micrometers, corresponding to the variation of the detection angle of less than 0.1°. The incidence angle of the RIs was also practically unchanged during the scan over the small sample area, which assured the proportionality between the number of SEs excited from the Pt plate and the number of RIs.

All measurements were performed in the chamber with a base pressure in the range of 10^−7^ Torr. The energy of the He ions was 35 keV with a beam current of 0.5 pA. The compensation of the sample charge by the electron flood gun was not possible due to the configuration of the sample holder.

The following model test samples were investigated: (1) a Au on carbon SEM test sample from Agar scientific, (2) an AFM standard TGQ-1 from NT-MDT: a grid of SiO_2_ square bars of 22 nm thickness on a silicon substrate, and (3) a mica (phlogopite) sample prepared by mechanical cleavage.

## Results

### Comparison of RI, SE and BSI imaging modes: Au on carbon test sample

**Figure 2:**
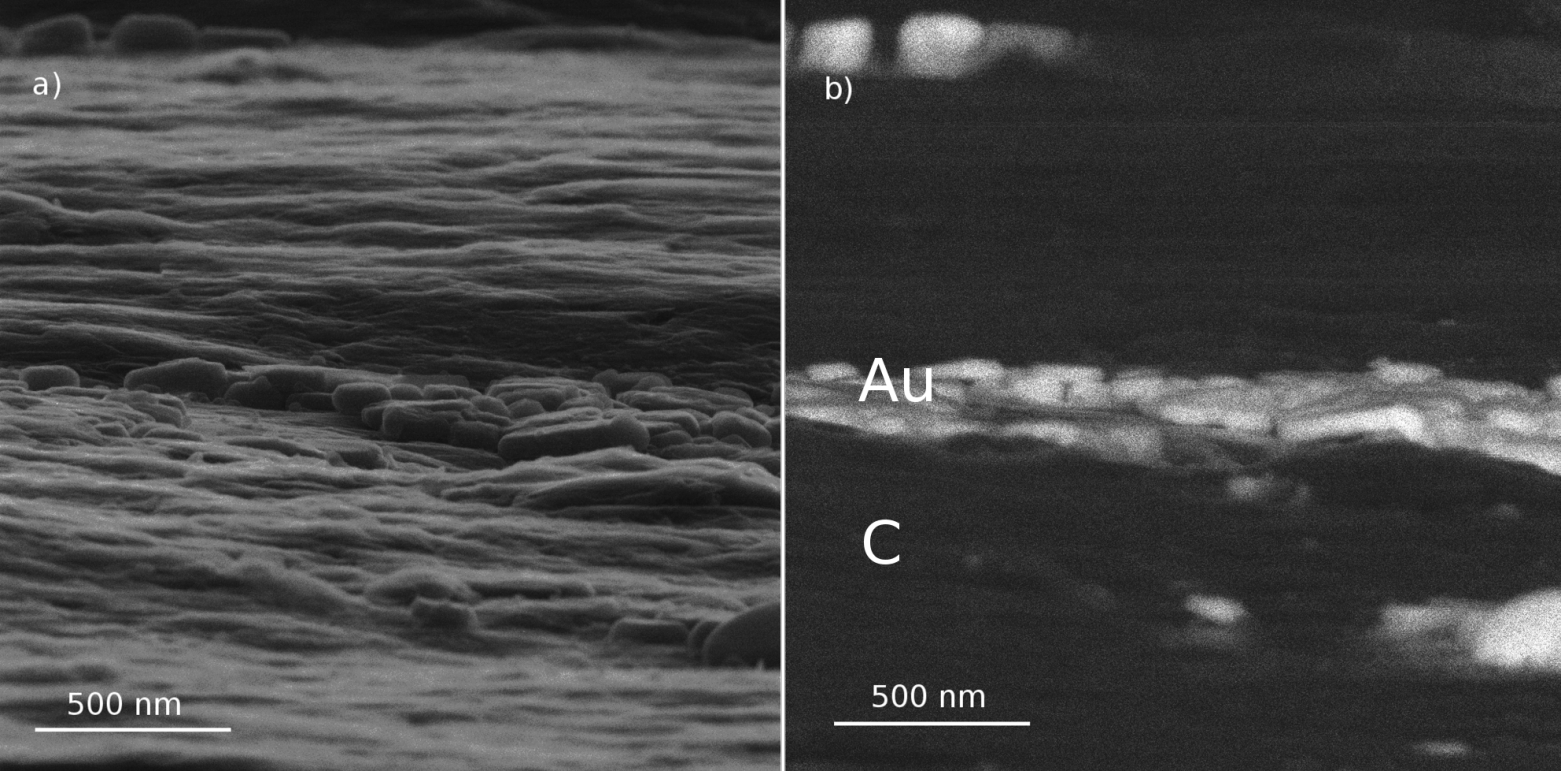
Images of Au on carbon by detection of reflected He ions (a) and backscattered ions (b).

Figure 2 represents the RI and BSI images of Au on a carbon sample taken at the grazing angle of incidence of 5° and the same angle for RI detection.

In Figure 2, one can easily recognize that only the central part of the images is in focus, which is due to the tilt of the sample with respect to the incident beam. This allows the independent estimation of the divergence angle of the He-ion beam from the pixel size and the depth of focus. The depth of focus is defined as the distance between the edges of the in-focused part of the image plane divided by the tangent of the grazing angle. From the image in Figure 2, the depth of focus can be estimated to be approximately 10 µm. Using digital signal processing in HIM, the image remains sharp unless the diameter of the beam is larger than the pixel size in the image. From the width of the image field of view (2.5 μm) and the number of acquisition points 1000 × 1000, the pixel size was calculated to be 2.5 nm. These values gave the angle of the beam divergence of approximately 0.5 mrad, which is in a good agreement with the manufacturer’s data.

Figure 2 represents the images of a Au on carbon sample that is partially covered by Au nanoparticles in the central part only. As expected, the Au particles exhibit a bright contrast in the BSI image on the dark background of the carbon substrate due to its significantly larger atomic number. On the contrary, the RI image does not show any noticeable signal difference between two different materials, giving information about surface morphology only. Additionally, the ratio of the signals from the top and side surface of the Au particles in the BSI and RI images are inverted. The top surface of the particles is brighter than the side wall in the RI image, whereas in BSI image, the situation is reversed.

**Figure 3:**
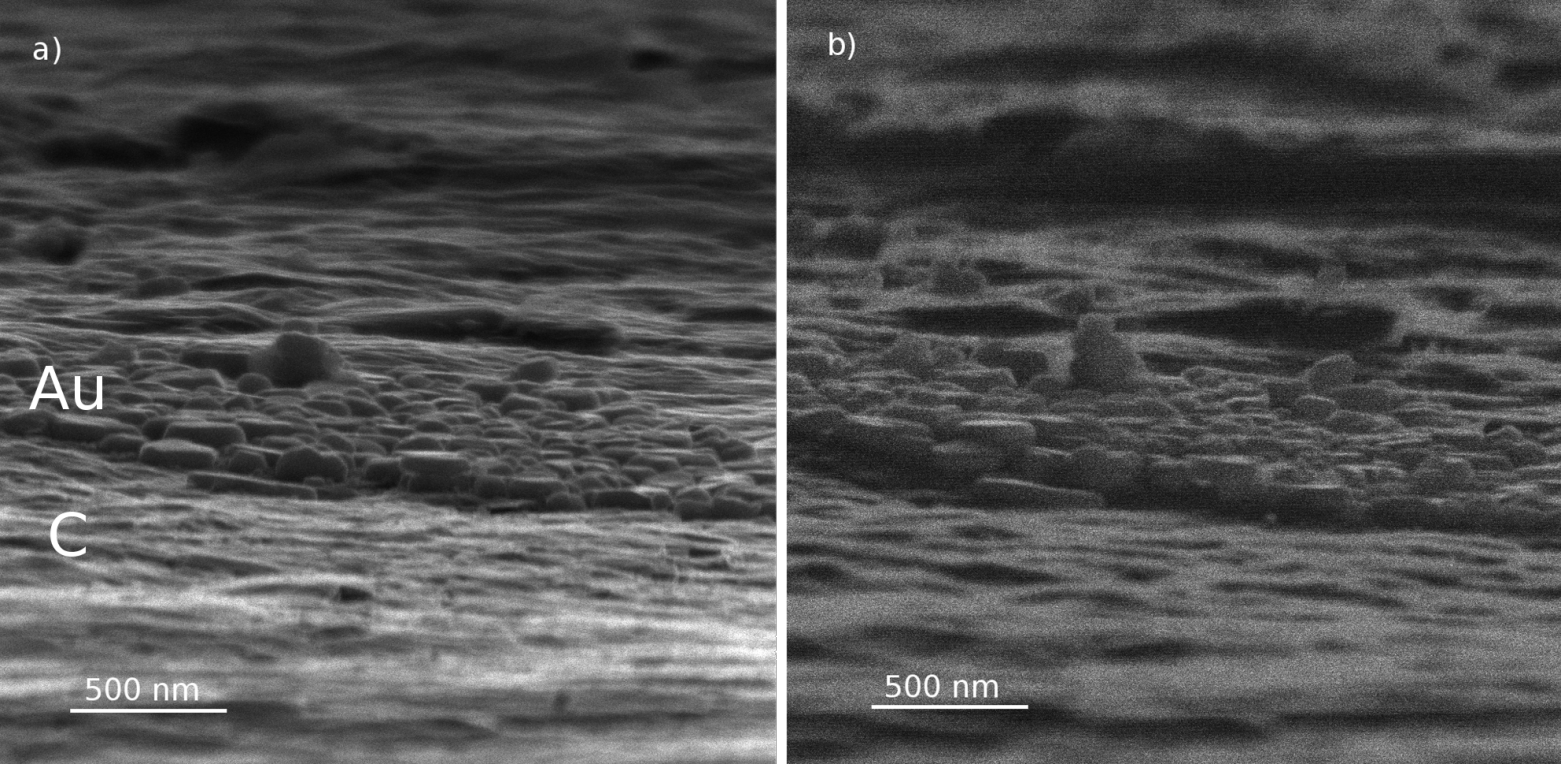
Images of Au on carbon obtained by detection of secondary electrons (a) and reflected He ions (b).

Figure 3a,b represents a comparison of the SE and RI imaging modes of Au on carbon. One can see that in both imaging modes there is no noticeable signal level difference between the Au nanoparticles in the central part of the image and the carbon substrate in the upper and the lower parts of the image. This implies that the RI yield at low ion incidence angles does not depend on the element properties and the RI contrast in the images originates from the surface morphology only in contrast to the case of normal incidence. One should also note that SE and RI images focusing on the same structural peculiarities differ from each other. A more detailed description of the difference will be treated in the next section.

### Reflection ion microscopy of silicon dioxide steps on silicon

**Figure 4:**
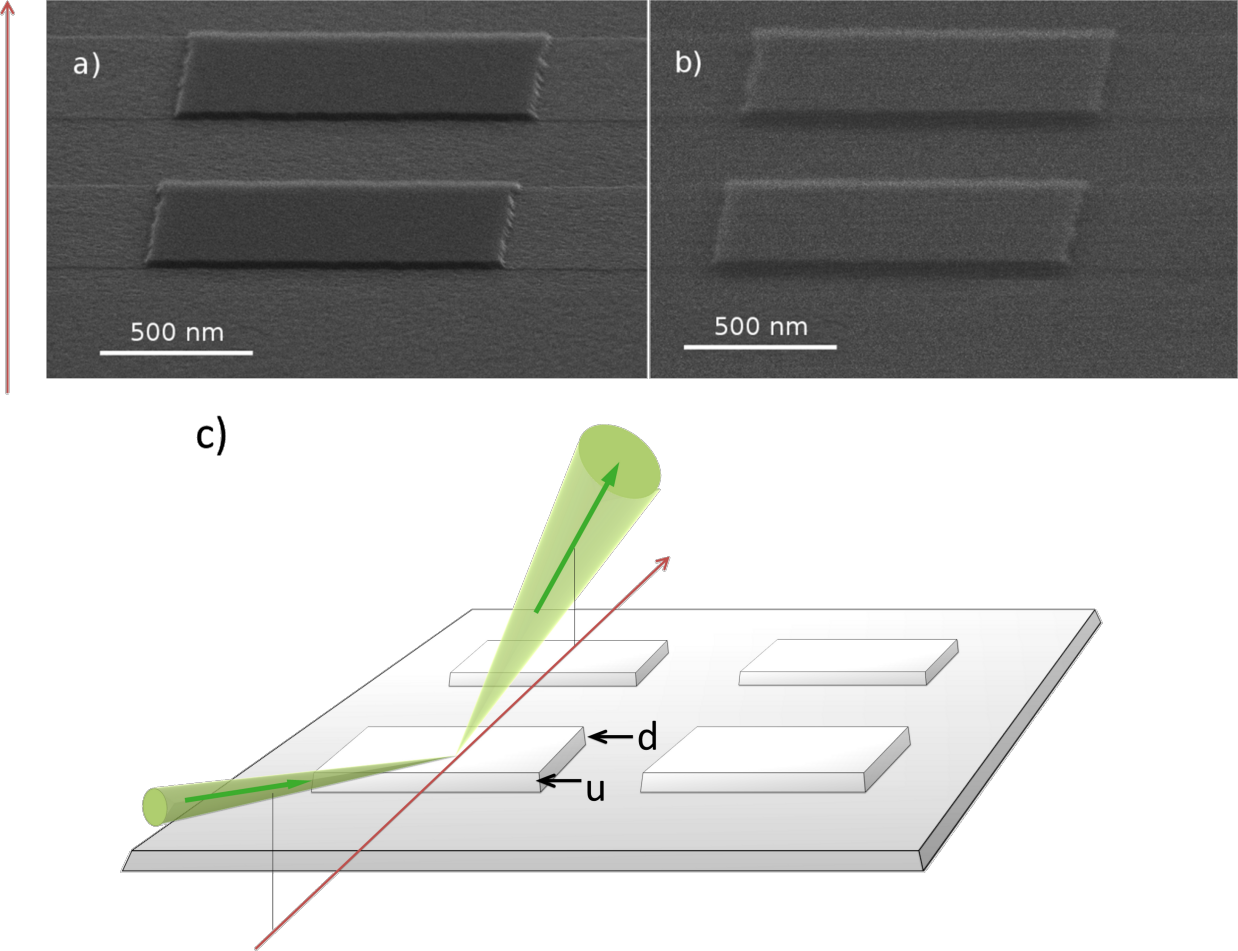
Images of silicon dioxide bars on silicon (TGQ-1 sample) obtained by detection of (a) secondary electrons, (b) reflected ions. (c) Sketch of ion beam path: green arrows correspond to the direction of the primary beam and reflected beam, the red axis corresponds to the vertical axis of the images in (a) and (b). “Upward” and “downward” steps are marked with “u” and “d”, respectively.

Figure 4 shows images of SiO_2_ bars on silicon obtained by the detection of SEs (Figure 4a) or RIs (Figure 4b) under the grazing and detection angles of 10°. One can easily recognize two main differences between the RI and SE images. Firstly, the contrast between silicon and silicon dioxide is clearly distinguishable in SE image, but not in the RI image. Note that the silicon dioxide bars are darker than the silicon substrate in the SE image (see Figure 4a), while there was no noticeable SE signal difference between Au and carbon (see Figure 3a). Additionally, the dark contrast of the upward steps (marked “u” in Figure 4c) seems to be broader in the RI image than in the SE image.

**Figure 5:**
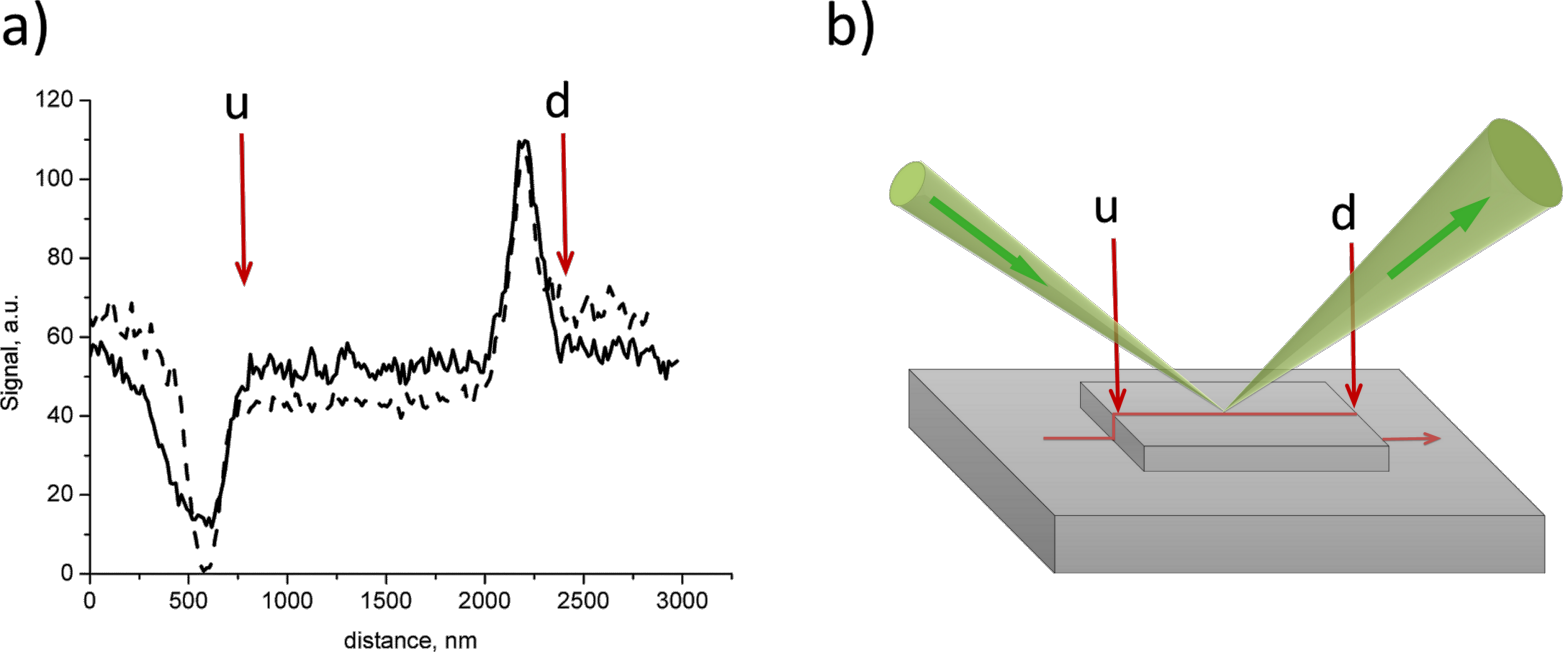
(a) Signal profiles: secondary electrons (dashed line) and reflected ions (solid line), red arrows shows the position of upward (u) and downward (d) steps. (b) Schematic view of ion reflection, green arrows correspond to the direction of the primary beam and reflected beam, the red line corresponds to the profile path, and the red arrows show the position of upward (u) and downward (d) steps.

The profiles of the SE signal and RI signal measured across the silicon dioxide bar are presented in Figure 5a. The profiles are measured along the beam projection, and the profiling path is shown by the red line in Figure 5b. The edge of upward step and the edge of downward step are marked with “u” and “d”, respectively. The comparison of the SE profile (dashed line in Figure 5a) and RI profile (solid line in Figure 5a) revealed that the dark area in the RI image of the upward step was broader than the dark area in the SE image for the same step. The width of the bright area in the RI image of the downward step was found to be equal to the width of this area in the SE image.

### Reflection ion microscopy of cleaved mica

**Figure 6:**
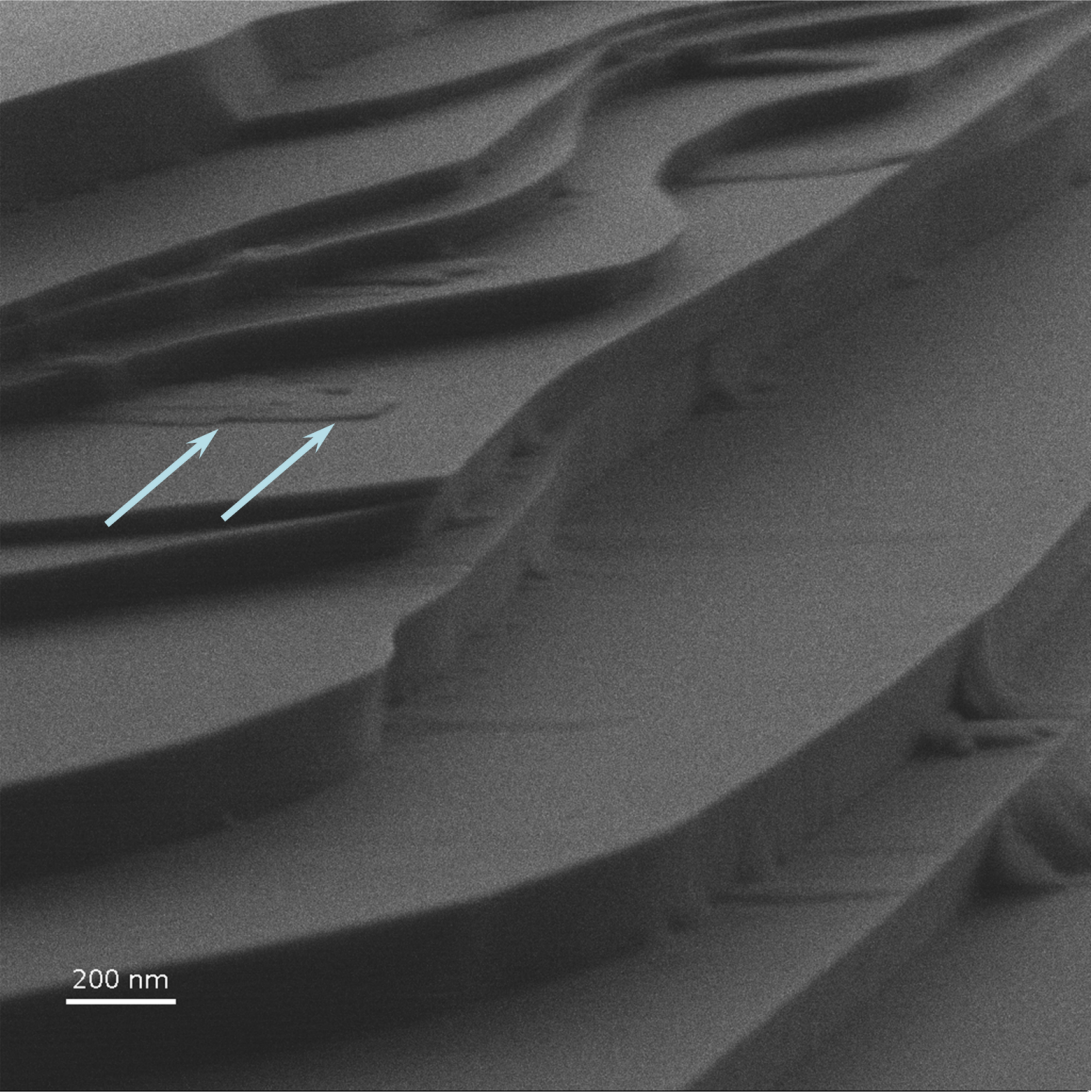
RIM image of cleaved mica. White arrows shows mark the smallest step in this image. Accelerating voltage: 30 keV, beam current: 0.3 pA.

Figure 6 shows an RI image of a cleaved mica surface obtained at a magnification of 100,000× under the grazing angle of 5° without charge compensation or conductive coatings. One can see that all details of the step-like surface are well reproduced without any noticeable image distortion that might be expected due to surface charging. Some distortion of the image caused by the charging appeared only at significantly higher magnification. It should be noted that the overall detector signal of registered electrons was not changed by the variation of the sample bias, indicating that SEs excited immediately near the sample could not reach the detector because of sample surface charging.

The step of a minimum height in the field of view of the image in Figure 6 is marked with arrows. The value of the height, as retrieved from the image processing, was found to be 7 ± 2 nm. The details of the calculation procedure will be described in the next section.

## Discussion

### Contrast formation in scanning reflection ion microscopy

The results presented in the previous sections showed that imaging using an incident ion beam at low grazing angles exhibits following properties:

- RI detection is insensitive to the atomic number or density of the material;
- there is a difference between SE and RI imaging of upward steps but not for the downward case;
- no distortion caused by charging effects is observed in an RI image for insulators.

An explanation for these RIM properties and an analysis of the advantages and limitations of RIM require a theoretical description of the ion reflection and detection processes. In this part of the paper such a description will be provided combining a simple geometrical modeling and ion–matter interaction simulations.

**Figure 7:**
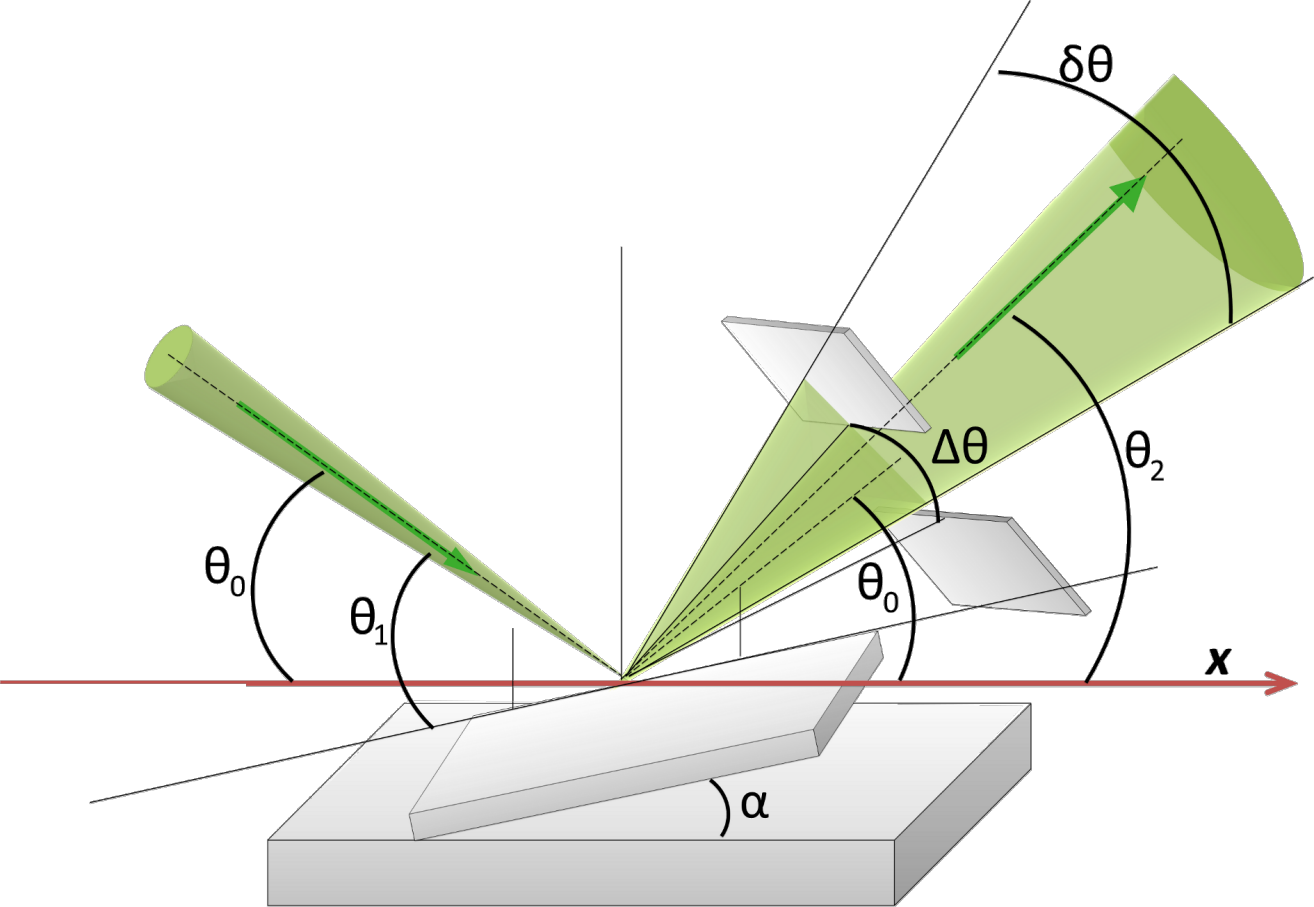
A schematic diagram of the incident and reflected ion paths with designations given in the text.

The following designations are used to describe the incident and reflection paths as shown in Figure 7: Θ_0_ - grazing angle between the incident beam and the specimen plane, Θ_1_ - angle between the incident beam and a local detail of the specimen surface, Θ_2_ - angle between the reflected beam and the specimen plane, ΔΘ - angular aperture of RI detection, δΘ - halfwidth of the angular divergence of RI, φ_1_, φ_2_ - polar angles of the incident and of the reflected beams, respectively, in the specimen plane (not shown in Figure 7). To describe the surface morphology we will use the angle α between the specimen plane and the detail of the specimen surface roughness under consideration. From this geometry, Θ_1_ = Θ_0_ + α. We will neglect the incident beam divergence angle since it is much smaller than both the angular aperture of the RI detector diaphragm and the halfwidth of the angular distribution of the RIs. Thus, we will further consider the incident beam as being infinitely narrow.

The number of reflected ions per incident ion is called the reflection coefficient or reflected ion yield. In the simple geometrical model, the reflection coefficient,η, depends on the angle of incidence and the reflection only, that is, η = η(Θ_1_,φ_1_,Θ_2_,φ_2_).

Firstly, we assume that the reflection coefficient is independent of the polar incident angle, φ_1_, that is, η = η(Θ_1_,φ_1_,Θ_2_,φ_2_) = η(Θ_1_,Θ_2_,φ_2_). This assumption is valid for amorphous materials and in the absence of ion channeling in crystalline materials. Ion channeling can cause the reflection coefficient to become strongly dependent on the angle in the vicinity of the channeling critical angles [28,30] and can be eliminated by a proper choice of the angle between the incident beam and crystal atomic planes [38].

The RI signal is the number of SEs per second measured by the ET detector as the flux of SEs from the Pt RI–SE converter (see (2) in Figure 1). The total number of reflected ions is proportional to the number of primary ions, *N*_I_, and the proportionality constant is the reflection coefficient, η(Θ_1_,Θ_2_,φ_2_). In fact, only those reflected ions that are transmitted through the slit aperture can reach the Pt-coated surface and excite the SEs. The portion of the RIs that reach the Pt converter is a function of the ion reflection angles *f*(Θ_2_,φ_2_). The number of SEs excited from th Pt surface is proportional to the number of ions reflected within the aperture, and the proportionality constant is the secondary electron yield for the Pt-coated surface, γ_0_. The SE yield depends on the angle of incidence and is determined by Θ_2_, thus, γ_0_ = γ_0_(Θ_2_). Thus, the detected RI signal should be calculated as the product of the number of reflected ions and the efficiency of SE detection, while integrating over all angles as shown in Equation 1.

[1]
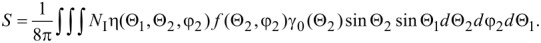


In the case of the slit-like aperture installed at a fixed angle, Θ_2_, one can assume that the portion of RIs that reaches the Pt converter is independent of the polar angle, that is, *f*(Θ_2_,φ_2_) = *f*(Θ_2_). This assumption gives rise to the explicit form of the *f*(Θ_2_) dependence: *f*(Θ_2_) = 1 if Θ_0_−ΔΘ/2 < Θ_2_ < Θ_0_+ΔΘ/2, otherwise *f*(Θ_2_) = 0.

The SE yield from the Pt surface depends on the ion angle of incidence with this surface, β, and can be approximated by the inverse cosine law [19]: γ(β) = γ_0_/cosβ. The typical ion grazing angle of incidence in our work was 5° and the slit angular aperture was 4°. In this case, β can vary between 6° and 14°. The cosine function varies only within 2% for this angular range and we can neglect dependence of the SE yield on the ion reflection angle, thus, γ_0_(Θ_2_) = γ_0_. We factor out the integral sign for the number of primary ions and SE yield and integrate Equation 1 over Θ_1_ and φ_2_ to arrive at the expression:

[2]
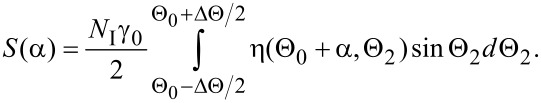


According to this expression, the RI signal is determined by the ion reflection coefficient and its angular dependence, the angular distribution of the reflected ions and the angular aperture of the slit diaphragm. We will now consider the effect of all these factors separately.

### Angular dependence of the ion reflection coefficient

**Figure 8:**
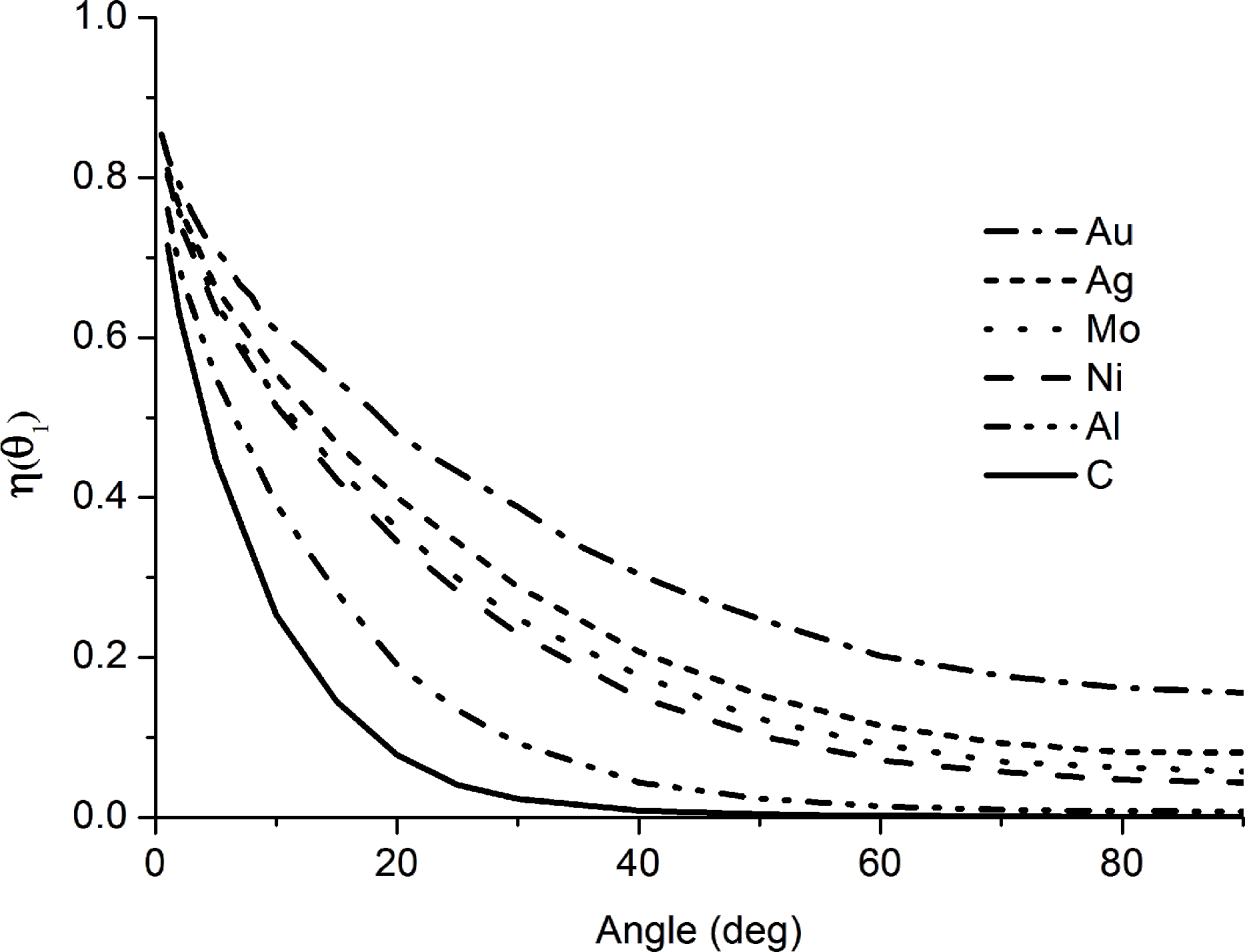
Dependence of the reflection coefficient of 35 keV He^+^ on the grazing angle calculated with SRIM software for different materials.

The dependence of the reflection coefficient for singly charged He ions on the grazing angle as obtained by a Monte Carlo simulation with SRIM software [39] for different materials is depicted in Figure 8. Note that the SRIM Monte Carlo simulation assumes that the ions are reflected from an amorphous target and all of them are collected by a detector.

As it can be seen in Figure 8, the ion reflection coefficient is a monotonically decreasing function that tends to the yield of backscattered ions when the grazing angle is greater than 90°. As Θ_1_ approaches zero, the reflection coefficient tends towards unity, implying that for reflection at very low angles nearly all ions will pass over the specimen surface. The highest relative difference in the reflection coefficient for the different materials (i.e., material contrast) is observed at normal incidence, whereas this difference reduces for low grazing angles and the material contrast vanishes. At the same time, the slope of angular dependence on the reflection coefficient is high at low grazing angles and low for normal incidence. This explains why the morphology contrast in the RI images is determined mostly by the surface morphology but not by the surface composition in our experiments.

In fact, the RI contrast is determined by the angular dependence of the reflection coefficient only when all of the reflected ions are detected. In turn, this assumption is valid when the halfwidth of the angular aperture is larger than the halfwidth of the RI angular distribution, that is, ΔΘ > δΘ, and the sample surface is sufficiently smooth so that |Θ_1_−Θ_0_| = α < ΔΘ. Under these conditions the expression for the RI signal given by Equation 2 can be simplified as:

[3]
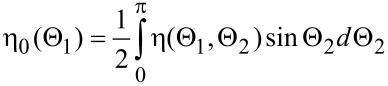


[4]
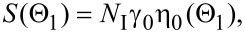


where η_0_(Θ_1_) is the RI reflection coefficient as a function of the grazing angle.

### Effect of angular distribution of reflected ions

As indicated above, the signal of the reflected ions is determined by the angle between surface and primary ion beam if all reflected ions are detected. In practice, some part of the reflected ions may be stopped by the diaphragm or by the elements of the specimen surface (shadowing effect). Firstly, we suppose that the angular distribution of the reflected ions η(Θ_1_,Θ_2_) has a maximum at an angle of Θ_2_^0^ that corresponds to specular reflection: Θ_2_^0^ = Θ_2_^0^ + 2α and the shape of the angular distribution of reflected ions is independent of the Θ_1_ angle.

The reflection coefficient can be presented as two multiplicands: η(Θ_1_,Θ_2_) = η_0_(Θ_1_) *g*(Θ_2_−Θ_2_^0^), where η(Θ_1_) is described by Equation 3 and *g*(Θ_2_–Θ_2_^0^) is the normalized angular distribution of the reflected ions, centered at Θ_2_^0^. Substituting this reflection coefficient into Equation 2:

[5]
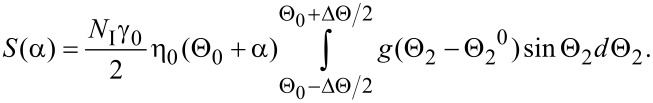


Using the notation *t* = Θ_2_−Θ_2_^0^ we obtain:

[6]
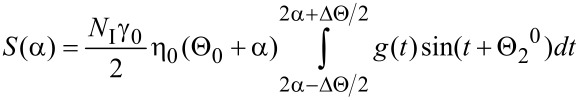


and variations of α can be described now as a shift of the integrating window. In particular, the comparison of Equation 4 and Equation 6 shows that the RI signal will decrease when α > ΔΘ.

Further, a suggestion for an explicit analytical shape of *g*(*t*) is required for the calculation of the RI signal profile with the help of Equation 6. If α > δΘ/2 + ΔΘ/4, then we can assume that the signal tends to zero because we integrate Equation 6 far from a maximum of *g*(*t*), that is, all ions are stopped by the diaphragm. Hence, such regions of the sample will appear in dark contrast in the RI image. Additionally, double (or multiple) reflections can take place giving rise to a “shadow effect”. This so-called shadow effect means that after the first reflection from the surface with low α, the ions are iteratively reflected from some pronounced details of the specimen and do not contribute to the signal. The simplest example of the shadow effect is the rectangular upward step that was investigated for the SiO_2_ sample bars on silicon (Figure 4).

**Figure 9:**
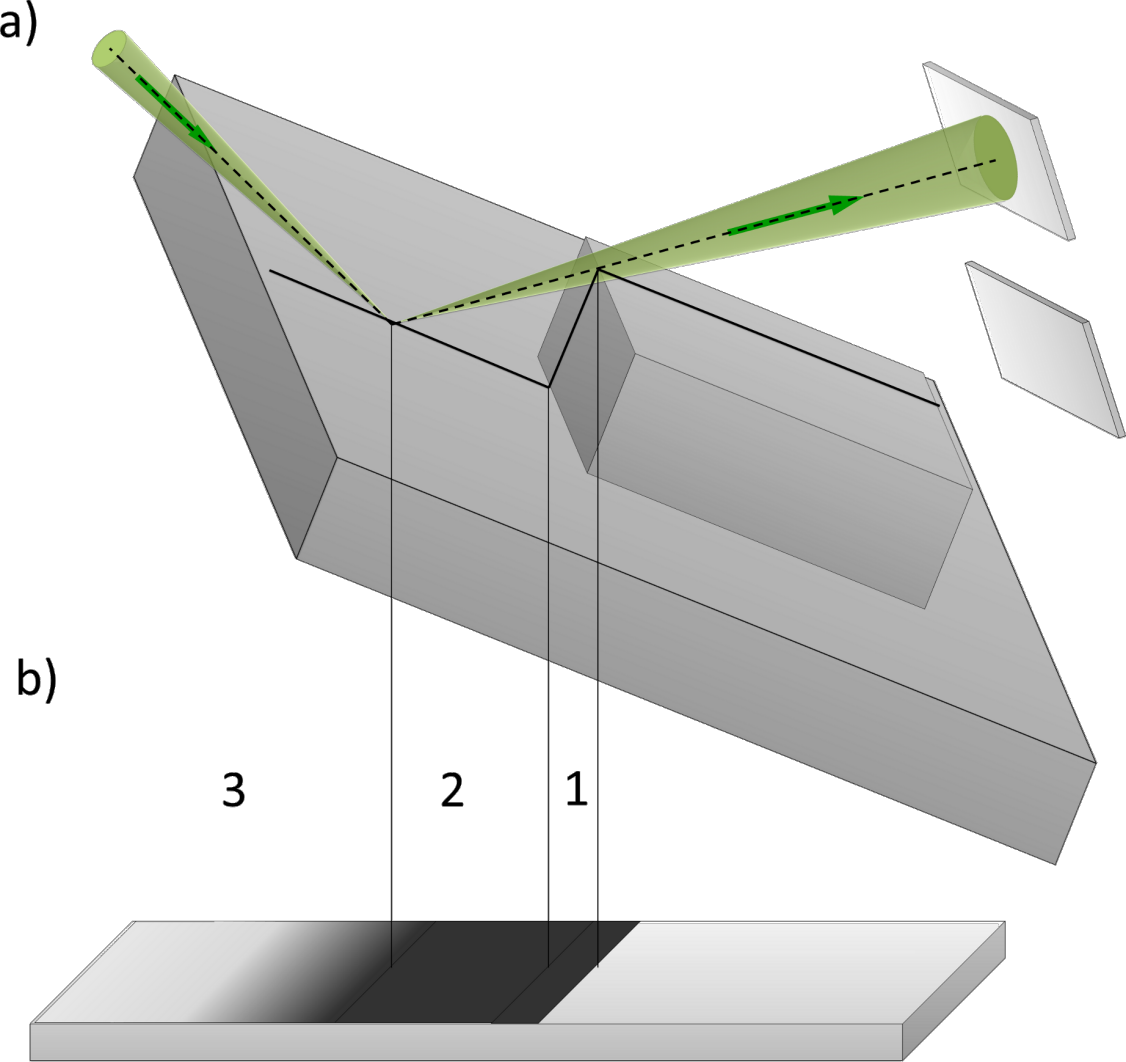
Designation of the regions of the upward step (a), and their projection onto the image plane (b): 1 – sidewall region, 2 – shadow region, 3 – penumbra region.

To further simplify the description, we assume that the surface between the steps is flat and parallel to the specimen plane (i.e., α = 0) and the step sidewall is perpendicular to the surface (i.e., α = 90°). The area at the step can be then divided into three regions, as shown in Figure 9.

In region 1 in Figure 9 all ions are reflected by the step sidewall away from the aperture and do not contribute to the detected signal, *S*(α) = 0. In this case, a dark area of width *d*_1_ = *h*cosΘ_0_ appears in the image, where *h* is the step height.

In region 2, all ions reflected from a plane bottom sample surface will be scattered by the sidewall, and an additional dark area (shadow) appears in the image (see Figure 9). If we denote the distance from step along the substrate as *x*, then the ions can pass through aperture when: Θ_2_ = arctan(*h*/*x*). In this case, the detected signal is described as follows:

[7]
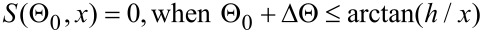


The width of the shadowed region 2, as calculated from simple geometry, is given by *d*_2_ = *h*∙sinΘ_0_/tan(Θ_0_ + ΔΘ).

In region 3 a smooth transition (penumbra) from zero RI signal to a constant RI signal from the substrate takes place. The form of the transition curve is determined by the RI angular distribution according to:

[8]
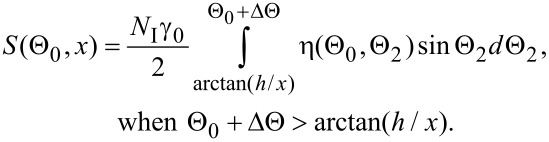


The total width of the dark area in the image of the step will then be a sum of the widths of regions 1 and 2:

[9]
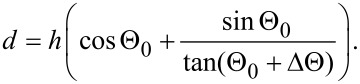


Thus, the step height can be calculated from the dark contrast width in the RI image and the halfwidth of the slit aperture as follows:

[10]
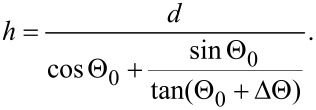


Using Equation 10 we calculated the step height from the experimental data presented in Figure 4 (Θ_0_ = 10°, ΔΘ = 2°, *d* = 40 ± 10 nm), where a value of *h* = 22 ± 6 nm was obtained, in good agreement with the AFM results.

In conclusion, the shadow effect and a finite aperture size result in dark areas in the image. These dark areas appears in the regions of a sample where α > δΘ/2 + ΔΘ/4. If a surface feature can be described with the rectangular step model, then Equation 10 can be used for the calculation of the step height.

### Effect of ion transmission

**Figure 10:**
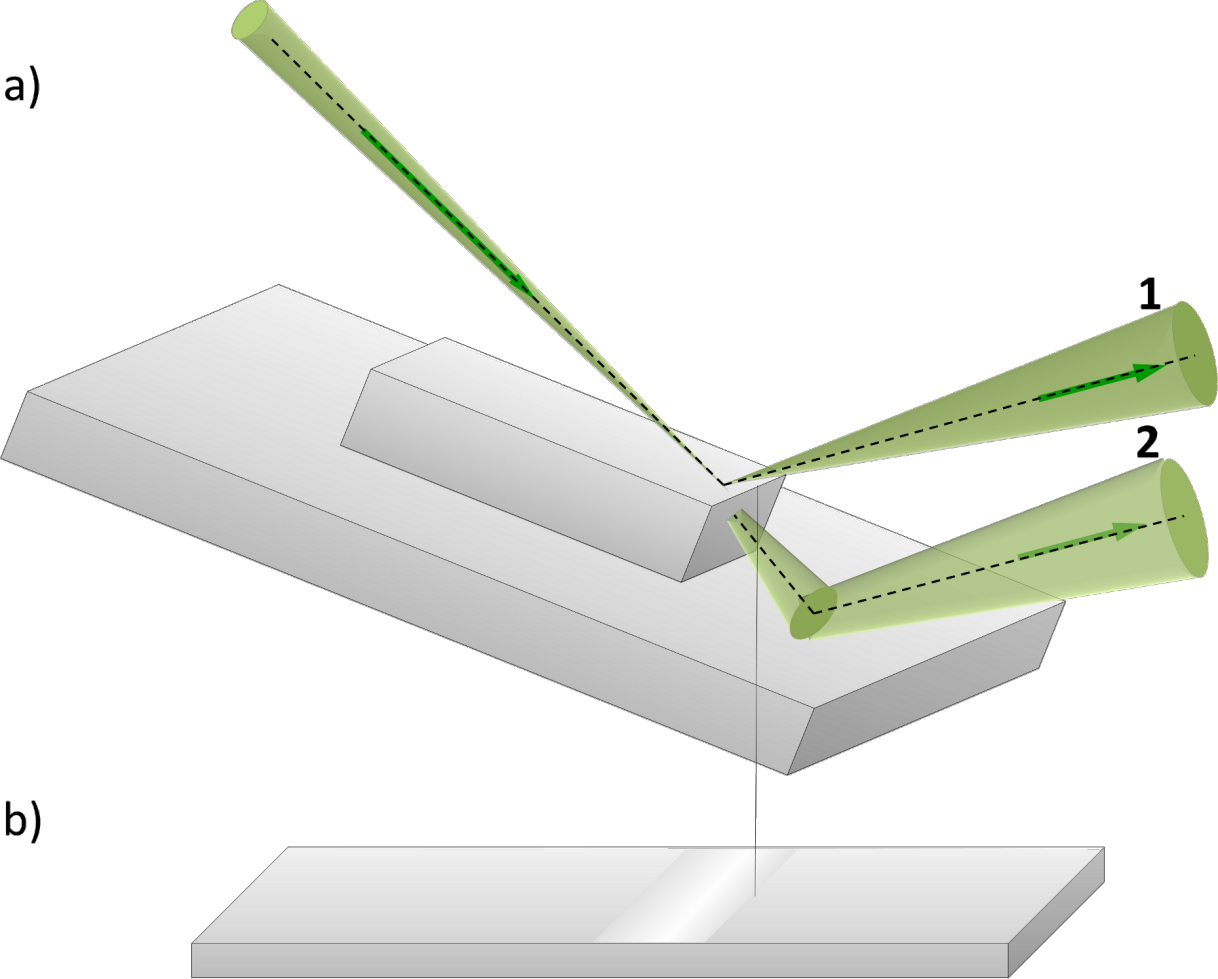
Ion transmission through the edge of a downward step (a) and its projection to the image plane (b).

The contrast formation mechanisms presented above describe the RI signal from the parts of the sample surface facing the incident ion beam. On the opposite side, the primary beam does not reach the surface and, accordingly, no RI signal can be obtained. In the vicinity of a sharp edge of a feature the ion transmission may contribute to the image contrast formation. As was demonstrated in Figure 4a, a bright contrast was observed in the downward step region. The peak position of the RI contrast coincided with one of the SE bright contrasts and with the sharp edge of the step. The bright RI contrast originates from the transmission of ions through the step edge and their subsequent reflection [40]. This divides the sample surface into two regions of reflection: the upper surface of the bar and the substrate surface beyond the step edge (see Figure 10a). To quantify the bright contrast profile we will use following simplified model.

The reflection coefficient of the primary beam from the upper surface (beam 1 in Figure 10a) is denoted as η^*^(Θ_1_,Θ_2_). The asterisk is used to emphasize that the reflection coefficient near the step edge differs from the reflection coefficient of the bulk sample surface when the distance to the step edge is comparable with the ion penetration depth. Near the step edge some part of the incident beam penetrates through it with the probability ρ(Θ_1_,Θ_3_) and hits the substrate at an angle Θ_3_, which we assume to be close to the angle of incidence, that is, Θ_3_ ≈ Θ_1_. The transmitted ions are reflected from the substrate with the reflection coefficient η(Θ_3_,Θ_2_) (beam 2 in Figure 10a). Using these designations the total reflection coefficient can be written as:

[11]
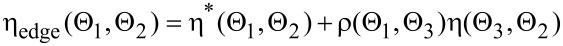


**Figure 11:**
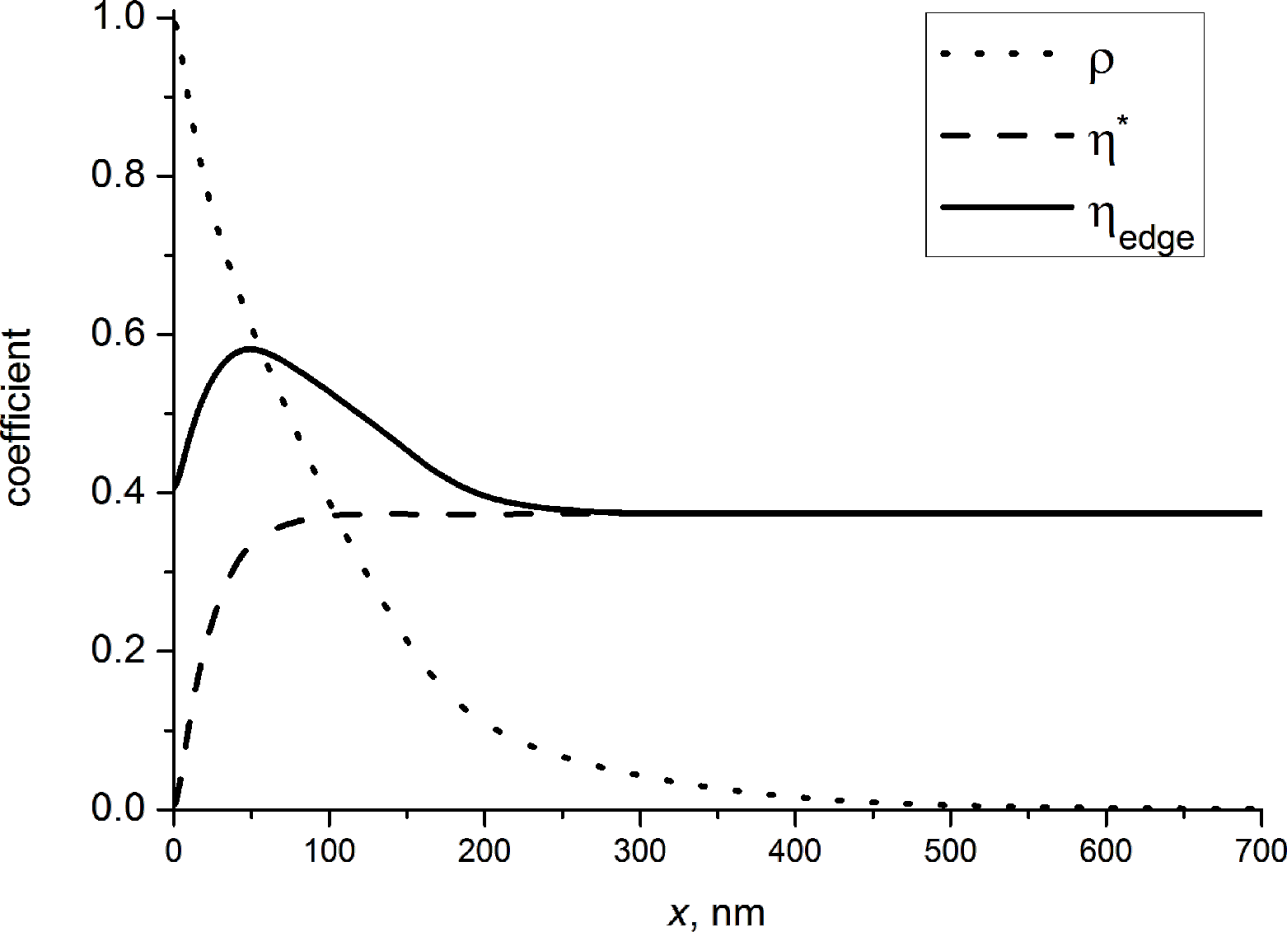
Dependence of the coefficients on the distance from the edge of the step: reflection coefficient from upper surface (dashed line), ion transmission probability (dotted line) and the total reflection coefficient calculated with Equation 11 (solid line) (angle of incidence was 10°).

Figure 11 shows the dependence of the reflection and transmission coefficients on the distance from rectangular step edge along the top surface. All values were calculated with SRIM Monte Carlo simulation software for a silicon dioxide step on silicon and 35 keV He ions. The ion transmission probability (dotted line in Figure 11) decreases from unity when the ion beam moves away from the step edge. On the other hand, the number of ions reflected from the upper surface (dashed line in Figure 11) increases with the distance from the step edge. The total reflection coefficient calculated using the Equation 11 (solid line in Figure 11) exhibits a maximum at a distance of about 50 nm from the edge of the step, which is related to the bright line in the RI image.

**Figure 12:**
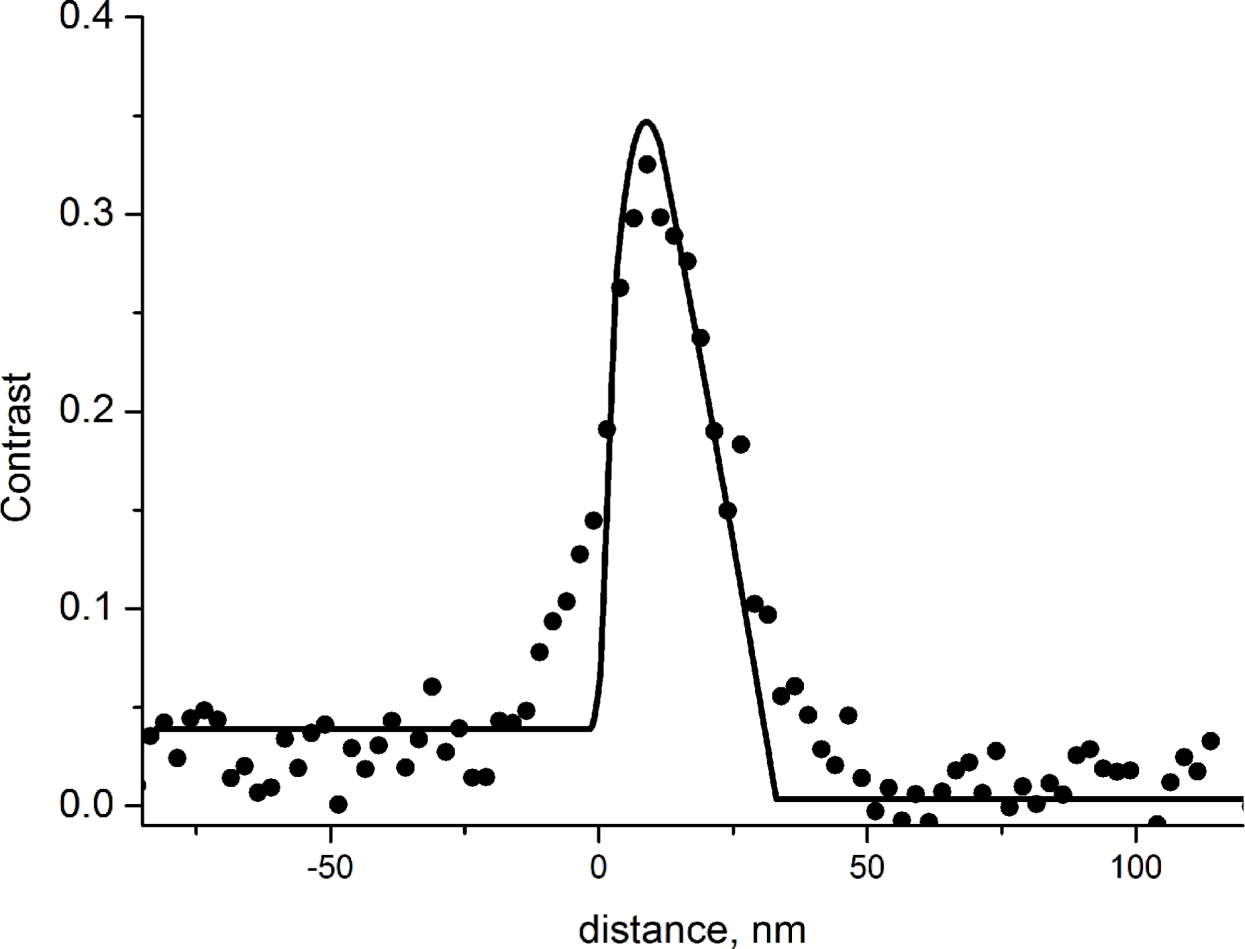
Profile of the relative contrast of a downward step: dots – experimental data, solid line – data calculated with SRIM software. Zero corresponds to the reflection from bulk SiO_2_.

The solid line in Figure 12 represents the profile of the RIM contrast calculated according to Equation 8 at a rectangular downward step of a SiO_2_ bar on Si. Far away from the step edge the reflection coefficient tends towards the constant value of the bulk material. The contrast is presented in Figure 12 with respect to the reflection coefficient value for a SiO_2_ bar.

From Figure 12 one can see that the simulated curve is in good agreement with experimental data, confirming the validity of the suggested simple theoretical model. A difference between the experimental points and the calculated results can be seen in the tails of the profile, which is caused by the finite size of the ion beam and the angular distribution of transmitted ions (assumed to be zero in the calculations).

From the point of view of applications of this technique, it is interesting to know the minimum value of the step height that can be distinguished in RIM contrast. Figure 11 shows that the edge contrast becomes negligible when the distance from the step edge is less than *x*_min_ = 10 nm. At the same time, the edge contrast can appear only when the primary beam projection passes above the bottom boundary between the step and the substrate. Consequently, the step height must be greater than *h* > *x*_min_tanΘ_1_ to observe edge contrast. In our case Θ ≈ 10°, and the edge contrast can be observed if the step height is greater than 2 nm.

In summary, the ion transmission through small features on the surface and their subsequent reflection can result in bright contrast in the RIM image. One should note that despite of the similarity of the bright contrasts in the SE and in RI images, the origin of their mechanisms are quite different.

### Effect of a surface charge

The RI image formation mechanisms described above originate from the ion scattering by the geometrical specimen relief and do not take into account the possible impact of surface charging produced by the positively charged ions. Our experiments with the mica surface demonstrated very sharp RI images without any noticeable effect of charging on the image quality, despite a strong positive surface charging that was revealed as the absence of the SE in the detected signal due to their attraction to the sample. Although the charging makes SE detection for imaging both at the normal and glancing ion incidence impossible to use, RI detection can still be successfully implemented.

Despite the fact that the energy of the SEs is three orders of magnitude less than the energy of the primary ions, the influence of surface charge on their trajectories might not be negligible, and the question regarding possible image distortion arises.

**Figure 13:**
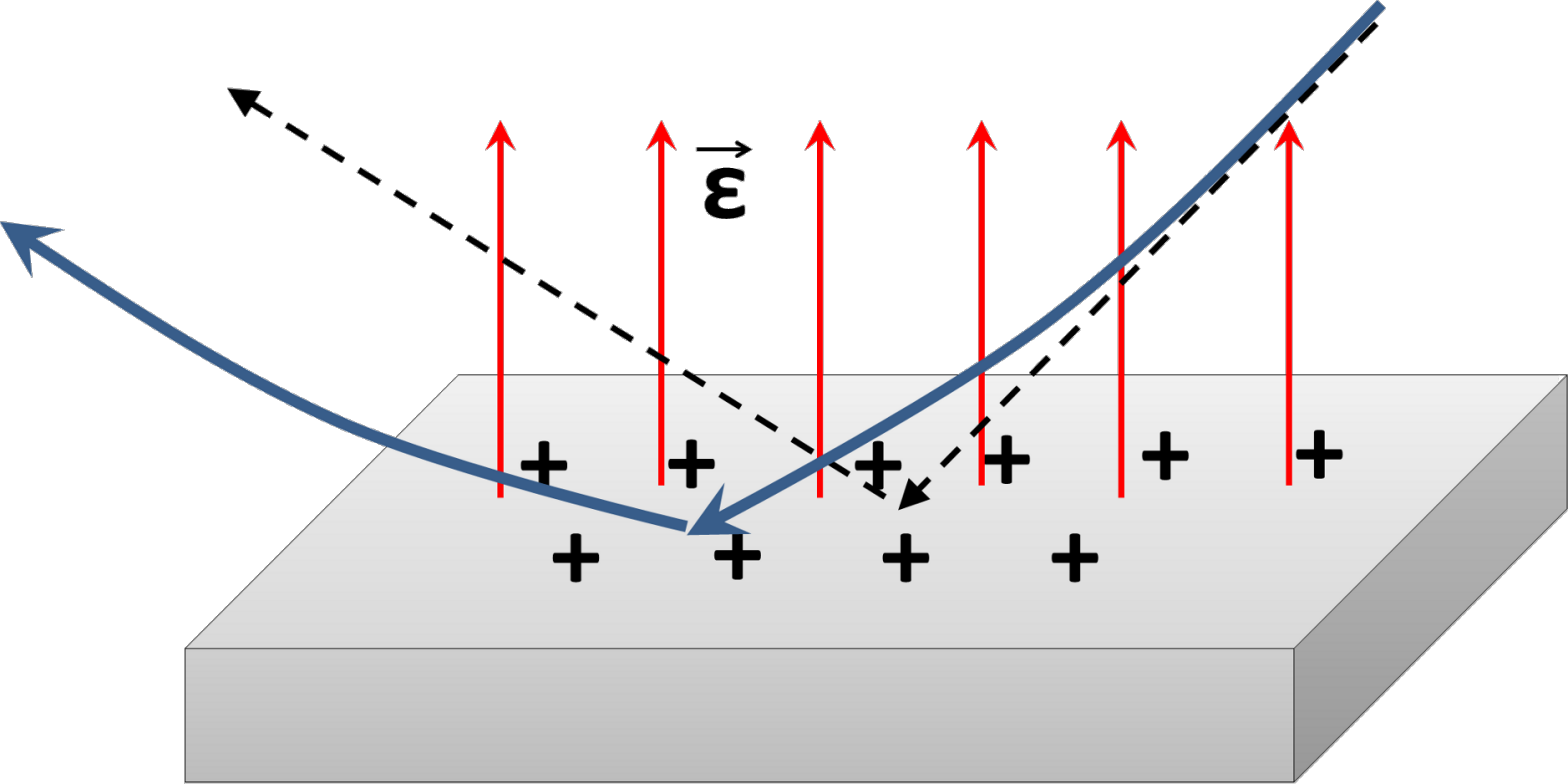
Reflection of ions from the charged surface. Trajectories of ions reflected from a charged surface (solid blue line) and trajectories of ions reflected from a neutral surface (dashed black line).

Under the action of the electric field of the positively charged sample surface, the trajectories of the primary ions become curved as schematically shown in Figure 13. The primary ion beam is decelerated before the reflection from the surface, but the reflected beam is accelerated back to its initial energy since the electric field is almost uniform.

In general, the grazing angle changes due to surface charging should be taken into account for the metrology of the surface relief. Theoretically, the changes of the angle can proceed until the beam is no longer parallel to the surface. Since that potential increases inversely proportionally to the size of the scan area with the same charge, the model predicts an increase of the charging effect on the beam trajectory with the increasing in the magnification. Accordingly, the simplest test to check for the presence of the effect of beam charge bending is a comparison of results of two scans preformed with different magnifications. Similar results will provide evidence of the accuracy of the lateral size definition. As for the accurate calculation of the step height from the shadow contrasts, it should be noted that the dependence of the width of the shadow on the angle of incidence (according to Equation 10) is rather weak for the experimental parameters used in RIM. In fact, a variation of the angle of incidence from 0° to 10° results in the relative variation of the shadow width of a few percent and does not affect the accuracy of the measurements.

In summary, reflection ion microscopy is useful for imaging of the surface of dielectric materials without the need for charge compensation, and the accumulated surface charge plays te role of positive sample bias. The impact of the charging on the height of the surface steps is negligible.

## Conclusion

In summary, we investigated the capabilities of a scanning reflection helium ion microscopy technique that was realized in a helium ion microscope for the first time. No additional RI detector was installed in the He IM chamber. Instead, reflected ions were detected with a conventional SE detector by using RI conversion to secondary electrons on a Pt-coated surface.

It was demonstrated that in contrast to the case of a backscattered geometry, the reflection coefficient of the ions, which are incident and reflected at low grazing angles, is not sensitive to the atomic number of the sample material, and the contrast in RI images is determined mostly by the surface morphology. This experimental fact is in agreement with the results of the Monte Carlo simulations of the angular dependence of the ion reflection coefficient. Accordingly, RIM can be used for the imaging of surface morphology but not for the imaging of surface composition.

The obtained experimental results show that there are some quantitative differences in the surface morphology imaging between SE and RI imaging modes. The quantitative calculation of the shadow contrast at a rectangular step was performed based on a simple geometrical model. The calculation results show that the width of the dark contrast area in the RI image of an upward step is approximately twice broader than the same step width in the SE image. At the same time, the width of a bright area is practically the same in the RI and SE image of a downwards step. It should be also noted that in contrast to RIM, the width of the dark contrast in the SE image can vary depending on the SE detector collector grid voltage. Accordingly, the precision of the RIM definition of the height of an individual surface rectangular step is noticeably better than with the SE detector. With regards to the subject of surface morphology, we should compare RIM with other methods of surface investigation such as REM and AFM.

Conventional REM demonstrates the capability of imaging of a single atomic step, and it is used mostly for this purpose. Single atomic steps were not observed by means of RIM yet, but there are several features of the developed detection system that limit the imaging capabilities of RIM. The REM imaging of surface steps exploits the specific diffraction conditions, and the obtained contrast is a superposition of phase contrast and diffraction contrast [9]. All described mechanisms of RIM contrast formation neglect the dependence of a reflection coefficient on the polar angle because a slit diaphragm was used in the experiment. In reality, this dependence contains additional information on the surface details [41], and the channeling effect and the deviation from channeling conditions can be used for RIM contrast formation analogous to diffraction conditions in REM. A 2D aperture instead of slit is required to limit both angles of the reflected beam. Another distinction of the REM setup is a high vacuum specimen chamber that is required for the observation of the surface steps. The high vacuum chamber is also required to reduce the effect of contamination as noted below. Thus, observation of atomic steps in RIM seems to be possible, but requires modification of the specimen chamber and detection conditions.

Atomic force microscopy as well as RIM allows for precise, quantitative measurements of the height of surface details; however, the scanning process is very slow. From this point of view, RIM has the same advantage as SEM: fast scanning and a large maximum field of view. On the other hand, scanning and measurements in AFM can be performed for all sides of the sample features, whereas in RIM (as well as in REM) only the side facing the beam is suited for investigation.

In this work, RIM was demonstrated to obtain sharp images of an insulator surface without the need for charge compensation or a conductive coating. A positive surface charge produced by ions attracts SEs but does not significantly change the RI trajectory and their detection. The changes in trajectory caused by the electric field of the charged surface can reduce the ion incident and reflection angles, but our calculation shows that their influence on the step height determination is negligible and can be monitored by changing the imaging magnification. The alternative method of the imaging of an insulating surface in HIM is charge compensation by an electron flood gun. Imaging with a flood gun requires accurate adjustment of the flood gun parameters and ion beam parameters to neutralize the surface charge. The scan speed decreases when line-by-line charge compensation is used. The reflected ions are less sensitive to surface charge than SEs, thus RIM of insulating surfaces is more straightforward. At the same time, RIM requires a special sample holder. One of the main disadvantages of RIM is the inability to image the whole surface of a sample. Part of a sample is always hidden by sample features. Thus, the potential advantage of RIM over the use of a flood gun is determined by the sample surface details. Unfortunately, the construction of the RIM specimen holder does not allow for charge compensation, and a direct comparison of these methods is impossible.

Beside the factors mentioned above, the sensitivity and the precision of the RIM measurements are obviously defined by the signal-to-noise ratio that increases with the image acquisition duration. There are two factors that limit measurement time in RIM: the ion beam induced deposition (IBID) of hydrocarbons and the sputtering of the surface material.

The first problem exists both in a case of RIM and in conventional HIM. Fortunately, IBID that is mainly stimulated by secondary electrons [21] can be eliminated in RIM by applying a positive bias to the sample and reducing the contamination growth rate, and it can be completely avoided by usage of high vacuum conditions.

Ion beam-induced sputtering always takes place and, unfortunately, the sputtering yield increases at low angles, and even for ions as light as He this can exceed one atom per ion. Thus, this effect is the main factor limiting the acquisition time. Most of the images were obtained with a dose of about 10^14^ cm^−2^, and the largest ion dose used for the acquisition of RI images of Au on carbon was about 10^15^ cm^−2^. In this case we expect the sputtering of a single atomic layer of the sample. The resolution of the obtained images was insufficient to observe such a sputtering effect, but it can be a limiting factor for the observation of the atomic steps. Thus, the further enhancement of the detection system is desirable to reduce the measurement time and to avoid sputtering of a sample.

In conclusion, reflection helium ion microscopy is a promising tool for the investigation of surface morphology and is especially useful when applied to non-conductive materials. Further development of this technique would require a special, more sensitive RI detector, higher vacuum conditions, and a polar angle limiting aperture.
